# Advancing internal exposure and physiologically-based toxicokinetic modeling for 21st-century risk assessments

**DOI:** 10.1038/s41370-018-0046-9

**Published:** 2018-08-16

**Authors:** Elaine A. Cohen Hubal, Barbara A. Wetmore, John F. Wambaugh, Hisham El-Masri, Jon R. Sobus, Tina Bahadori

**Affiliations:** 1 National Exposure Research Laboratory (NERL), US EPA, Washington, USA; 2 National Center for Computational Toxicology (NCCT), US EPA, Washington, USA; 3 National Health and Environmental Effects Laboratory (NHEERL), US EPA, Washington, USA; 4 National Center for Environmental Assessment (NCEA), US EPA, Washington, USA

**Keywords:** PBPK modeling, Exposure modeling, Epidemiology

## Abstract

Scientifically sound, risk-informed evaluation of chemicals is essential to protecting public health. Systematically leveraging information from exposure, toxicology, and epidemiology studies can provide a holistic understanding of how real-world exposure to chemicals may impact the health of populations, including sensitive and vulnerable individuals and life-stages. Increasingly, public health policy makers are employing toxicokinetic (TK) modeling tools to integrate these data streams and predict potential human health impact. Development of a suite of tools for predicting internal exposure, including physiologically-based toxicokinetic (PBTK) models, is being driven by needs to address large numbers of data-poor chemicals efficiently, translate bioactivity, and mechanistic information from new in vitro test systems, and integrate multiple lines of evidence to enable scientifically sound, risk-informed decisions. New modeling approaches are being designed “fit for purpose” to inform specific decision contexts, with applications ranging from rapid screening of hundreds of chemicals, to improved prediction of risks during sensitive stages of development. New data are being generated experimentally and computationally to support these models. Progress to meet the demand for internal exposure and PBTK modeling tools will require transparent publication of models and data to build credibility in results, as well as opportunities to partner with decision makers to evaluate and build confidence in use of these for improved decisions that promote safe use of chemicals.

## Introduction

The increasing demand to replace traditional toxicity testing with more efficient, non-animal approaches has fueled rapid advances in toxicology and exposure science [1]. New toxicity screening approaches that measure molecular and cellular responses following chemical exposure span a range of complexities, from cell-free reporter-based assays to cellular and organotypic systems designed to recapitulate molecular and cellular perturbations that can in turn inform mode of action or adverse outcome pathway assessments [2–4]. In parallel with these efforts, appropriate internal exposure-dose modeling tools are required to bridge these in vitro potency data to an appropriate in vivo exposure metric [5]. Consideration of decision context as well as physiology, target tissue, and pharmacokinetics are required for success in such internal exposure modeling. Physiologically-based toxicokinetic (PBTK) modeling encompasses all of these factors systematically, providing a critical approach for these 21st century risk assessments [6–10].

**Fig. 1:**
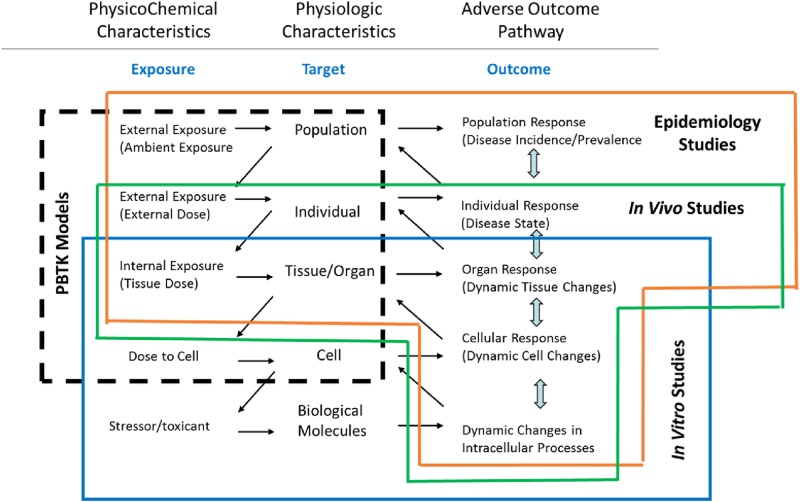
Physiologically-Based Toxicokinetic Model (PBTK) coverage from exposure to target dose and across levels of biological organization. Associated coverage of in vitro bioactivity, in vivo toxicology, and epidemiology studies

PBTK modeling employs a compartmental structure that incorporates anatomic and physiologic characteristics of the body and its tissues to map chemical movement [11]. These models have typically been used to translate and extrapolate results from traditional animal toxicology studies to inform understanding of potential impacts in humans for a variety of chemical safety evaluations. However, the organizational framework of PBTK models is equally well-suited to incorporating pharmacokinetic data from in vitro or in silico data streams for use in in vitro-in vivo extrapolation (IVIVE) [8, 10]. Similarly, the flexibility to modify this framework with either increasing or decreasing complexity makes PBTK modeling amenable to explore many scenarios of relevance to toxicologists, exposure scientists, and epidemiologists alike (Fig. 1). As these fields adopt alternative and higher throughput experimental and computational methods to collect data on chemical impacts in biological systems, advances in PBTK modeling, as well as biomonitoring to inform exposure reconstruction, are required. These advances will enable exposure-response characterization across new test systems, evidence integration across studies, and information to address the most pressing public health decisions. Here we offer some examples of how advances in internal exposure modeling and biomonitoring approaches are meeting the demand for model development, model evaluation, and data generation to inform future chemical risk evaluation.

## Applying PBTK modeling tools to support risk-informed public health decisions

Physiologically-based pharmacokinetic (PBPK) and/or PBTK models are based on the premise that xenobiotic concentrations in the plasma or target tissue can be quantitatively predicted given consideration of an external dose or exposure measure, anatomy and physiology of the system under study, and the underlying pharmacokinetics or toxicokinetics of the xenobiotic [12]. PBPK models have been used by the pharmaceutical industry to predict delivery of therapeutics to target tissues as well as to determine the potential for drug–drug interactions [13–15]. A clear separation between the system-level information, xenobiotic information and the dosing or exposure scenario has extended the application of PBPK and PBTK models to incorporate estimates of human variability, providing a more comprehensive evaluation of these agents [16–19]. Moreover, documentation of the model design, input parameters, and the underlying differential equations used to calculate the tissue concentrations provide a needed transparency for model evaluation.

Although model architecture (i.e., chemical, physiological, and mathematical description) is the same for both PBPK and PBTK, PBPK modeling focuses on therapeutic compounds while PBTK modeling is employed to assess environmental chemicals [20]. The applications are somewhat different in that the chemical space for non-therapeutic chemicals may be more diverse [21], requiring consideration of absorption, distribution, and metabolic processes not necessarily common to therapeutic drugs. Also, the range of internal exposure levels may vary widely, requiring consideration of different chemical behavior (e.g., linear, non-linear) in the models. PBTK models are often used in conjunction with environmental chemical toxicity studies typically conducted using an experimental animal model to facilitate extrapolations from these studies to predict the external dose or exposure for the species of interest (i.e., humans or wildlife) from chemical concentrations measured in the model system [22]. Additional extrapolations (i.e., high-to-low dose [7], route-to-route [23]) may also be necessary to estimate human risk depending on the data available. Alternately, reverse dosimetry application of PBTK models can infer exposures that either (1) would be consistent with biomarkers measured in biological media [24] or (2) would cause concentrations at an internal site of action found to be adverse in in vivo-based or in vitro -based experiments [25]. These estimated external environmental exposures can then be the focus of risk-informed decisions and actions [1].

In 21st century risk evaluations, the decision context will drive the required internal exposure modeling tools by framing the questions and bounding the certainty required from model predictions [6]. Development of PBTK models has typically relied upon animal-testing, but there are thousands of chemicals in commerce for which animal testing has never been conducted [26]. For some decisions requiring rapid assessments across a large chemical space, data-derived extrapolations may suffice to guide next steps [5]. Other decisions may require a complex PBTK model to understand variability in physiologic response, explain unusual chemical-specific physiological processes, predict potential impacts, and/or take public-health protective action. Initial PBTK modeling and subsequent model refinement will be guided by both this context and available data [27].

## Examples of recent advances in PBTK modeling tools

Current model development focuses on broadening the utility of PBTK for risk-informed chemical evaluation. These advances include: higher-throughput evaluation of hundreds of data poor chemicals; strategies for predicting impacts to susceptible lifestages; and approaches for addressing exposures to real-world mixtures. In addition, methods for using PBTK models to estimate external exposures from biomonitoring data have progressed.

To facilitate interpretation of high-throughput screening and in vitro bioactivity data, as are being generated in the US EPA ToxCast and US Tox21 programs, the nominal testing concentrations at which in vitro bioactivity is observed must be related to an external exposure required to achieve similar internal exposures. In addition, nominal exposure itself must be related to free concentration in vitro [28, 29]. Traditional PBTK modeling approaches where in-depth studies are conducted on one chemical at a time to ascertain relevant behaviors (including chemical biotransformation pathways, dose-response, target tissue distribution, exposure pathways, and modes of action) are challenged to meet the needs for applying these high-throughput data streams to support risk-based evaluations for large sets of chemicals.

To address this problem, a simplified in vitro–in vivo extrapolation (IVIVE) approach developed for pharmaceuticals [16] was adapted to chemicals in the environment [25, 30]. This approach integrates in vitro TK data along with computational modeling to predict internal exposures that include quantitative estimates of population variability [25]. In keeping with the high-throughput nature of the ToxCast dataset, Rotroff et al., identified key determinants of chemical TK and measured these experimentally to provide a prediction of what an internal blood concentration would be, given a repeated daily exposure (mimicking a chronic daily exposure to chemicals). A simple compartmental model was used, and the predicted plasma concentrations were assumed to be representative of the target tissue concentrations. Any considerations beyond hepatic metabolic clearance, blood binding, and non-metabolic renal clearance were either neglected (e.g., extrahepatic metabolism) or set to conservative assumptions (e.g., 100% oral absorption). This simple approach provided a foundation that is actively undergoing refinement to incorporate additional information and outputs. These  include prediction of additional parameters and outputs (e.g., *C*_max_) [31], additional routes of metabolism, as well as further exploration of population variability and identification of sensitive populations.

Recently, a follow-up effort based on the Rotroff et al. [25] approach was utilized to assess the range of TK variability that may be anticipated across different populations. Differences in physiology, genetics, and development (i.e., ontogeny) can lead to vastly different internal chemical concentrations following external exposure to an identical amount of chemical. Through application of Monte Carlo modeling approaches, in vitro data, physiologic, ontogenetic, and genetic differences across different populations can be integrated to quantitatively predict the internal concentrations across different lifestages and populations [32]. Initial efforts in this area have revealed anticipated ranges of TK variability that may result, and can potentially identify key factors that drive the variability as well as a potential tiered strategy to decide which chemicals may require follow-up characterization to better understand the extent and implications of this variability. In addition, these efforts demonstrate the possibilities available to incorporate a range of tools and approaches (in vitro, in silico, etc.) to inform the task at hand.

As noted above, approaches for IVIVE that utilize high-throughput screening data are usually based on predictions for an oral equivalent dose that will produce steady-state blood levels comparable to the high-throughput in vitro assay concentration where half-maximal effects are observed (i.e., AC50) [33]. Such an assumption is appropriate when blood levels are reasonable surrogates for target tissue levels, and steady-state blood levels are attained quickly for chemicals with short half-lives. However, external dose (external exposure) equivalents estimated using reverse dosimetry based solely on steady-state blood levels is inadequate in situations where target-tissue levels are different from blood, perhaps due to accumulation of a chemical because of specific protein binding, deposition in the lipid phase, or active membrane transport kinetics. In these cases, the interactions between ADME, target tissues, and toxic response are qualitatively described using adverse outcome pathways (AOP). AOPs provide a biologically-based framework for linking molecular initiating events (MIEs) triggered by chemical exposures to cellular key events (KEs) leading to adverse outcomes (AOs) [34]. This AOP framework facilitates links between the external exposure that is the focus of risk managers and the internal exposure or target tissue dose associated with adversity [35]. Application of data generated from high-throughput in vitro assays and AOPs to estimate health risks from exposure to environmental chemicals during sensitive windows of development is additionally complicated by changing life-stage TK determinants.

Computational strategies that consider the impact of lifestage, physiology, and biochemical changes on toxicity potential have demonstrated the applicability of this approach to provide mechanistic insight into linkages between maternal exposures and predicted responses in the embryo and infants. In one, a human lifestage PBTK model was developed to quantitatively describe chemical disposition during pregnancy, fetal development, neonate, and child growth stages. Maternal exposures were estimateded that would yield fetal blood levels equivalent to the chemical concentration that altered in vitro activity of ToxCast assays for critical vascular signaling targets described in a developmental toxicity AOP [36]. The resulting in vivo oral dose estimates were then compared to lifetime exposure levels using literature data or exposure models to derive an AOP-based margin of exposure (MOE), providing a critical risk-related context to the data [18]. In another example, an exposure modeling framework integrated with a lifetime PBTK model was employed to estimate corresponding external and internal systemic doses of bisphenol A and metabolites with a focus on gestational and neonatal developmental stages. Toxicity biological pathway altering doses for bisphenol A [33] were used to provide an alternate internal reference dose, feeding into another useful application of high-throughput screening data to inform an MOE-based approach [37].

Simple vertebrate systems offer additional opportunities to interrogate for AOs during critical windows of susceptibility. Zebrafish embryos provide an economical and higher throughput experimental model to screen chemicals for developmental toxicity potential [38] compared to traditional mammalian systems. Several examples in the literature illustrate the use of zebrafish embryos to study the effect of chemicals on gene and protein patterns and the potential implications of differential expression for toxicity [39]. Examination of chemically induced AOPs leading to disruptions of embryonic development can then enable estimation of quantitative internal exposure-response relationships [40] through incorporation of this experimental data and information on systems-specific modulators into a PBTK modeling framework [41].

PBTK models of critical windows can also be applied to inform interpretation of epidemiological data. Verner and coworkers [42] used a PBTK model of pregnancy to better understand potential impacts of prenatal exposure to perfluoroalkyl substances (PFAS) and potential confounding. The authors postulated that some of the association of PFAS exposure with lower birth weight seen in epidemiologic studies could be attributable to glomerular filtration rate (GFR). Simulated population estimates were compared with those from a meta-analysis of epidemiologic data. The resulting simulations suggested that a substantial proportion of the association between prenatal PFAS and birth weight may be attributable to confounding by GFR. This modeling approach was also applied to identify the period during pregnancy when this confounding is likely to be most pronounced. Strategies such as this one for incorporating PBTK tools to evaluate epidemiological information, have the potential to provide key mechanistic insights, improve study design, and further application of study results to improve public health decisions.

At the same time, that advances are being made in PBTK modeling for higher throughput applications, the availability of physiological and biochemical data from a variety of species has enabled the development of increasingly more complex and accurate PBTK models to computationally represent biological systems and provide more refined estimates of internal exposure. These advances are clearly illustrated by the use of PBTK modeling to quantitatively assess toxicological interactions between chemicals in complex mixtures. People are often exposed to complex mixtures of environmental chemicals such as gasoline, smoke, water contaminants, or food additives. However, investigators have often considered complex mixtures as one lumped entity in experimental setups. Valuable information can be obtained from these experiments, though this simplification provides little insight into the impact of a mixture's chemical composition on toxicologically-relevant metabolic interactions that may occur among its constituents. To that extent, PBTK models provide a quantitative format to address the impact of physiological and biochemical interactions on disposition of chemicals in biological tissues and its linkage to toxicological response. In one early example, Haddad et al. [43] applied PBTK model for the quaternary mixture BETX (benzene, ethyl- benzene, toluene, and xylene. Their BTEX model was the basis of several other approaches including application of Marko Chain Monte Carlo methods, and integration with toxicological endpoints for the health risk assessment for mixtures [44]. More recently, Jasper et al. [45] developed an approach that applies chemical engineering lumping methods to complex mixtures, in this case gasoline, based on biologically relevant parameters used in PBTK models. Using a rat inhalation exposure model, experimental time-course kinetic profiles of ten target chemicals in blood (of 109 identified in the exposure chamber) were compared to simulated blood levels with various numbers of lumps in a general PBTK model. Simulation error was significantly reduced by incorporating enzymatic chemical interactions and by lumping the 99 non-target chemicals. This biologically-based lumping method provides a systematic data and modeling-driven strategy to simplify the gasoline mixture while preserving the interaction effects of the entire complex mixture [45]. These studies demonstrate key ways PBTK modeling can identify critical data needs and provide solutions for understanding impacts from real-world, complex exposures, which ultimately can serve to promote holistic public health decisions.

Long valued for the potential to link chemical exposure to a biologically active dose [46], applicability of human biomonitoring data in risk assessment is often limited in non-occupational scenarios where external exposure information and scenarios are typically lacking. Recent efforts in exposure reconstruction, where PBTK modeling and statistical approaches are incorporated with biomonitoring data to estimate external exposure (also known as reverse dosimetry), have made great strides. Several sophisticated approaches have incorporated statistical methods to evaluate the uncertainty and variability that may be introduced given available input data and the use of simplifying model assumptions. For 21st century risk assessments where a balance between speed and precision in model development is an important consideration, one recent effort compared the impact of iterative and non-iterative approaches on precision and identified parameters necessary for a more accurate exposure reconstruction for short half-life chemicals, where interpretation of biomonitoring data is particularly problematic [47]. In another, use of a nested Monte Carlo simulation in reconstruction of acrylonitrile exposure estimated the range of uncertainty in the exposure concentrations and identified several metrics including exposure duration and certain physiologic parameters that could have a dominant influence on model outcomes [48]. Finally, McNally and co-workers employed an extensive, computational framework that integrated PBTK modeling, global sensitivity analyses, Bayesian inference, and Markov Chain Monte Carlo simulation in an approach to that could be used to reduce the dimensionality of certain reconstruction efforts with a minimal loss of precision [49]. Integrated strategies such as these will be critical in understanding the dependencies of reconstruction model performance on the type and extent of biological detail incorporated.

## Path forward

Recent advances in PBTK and other internal exposure modeling approaches and tools are beginning to bridge high-throughput in -vitro bioactivity data, mechanistic insights from complex experimental models, and traditional toxicity data with information on exposure and epidemiology to build understanding from these multiple lines of evidence. Continued progress in PBTK modeling tools to address pressing public health questions on chemical safety require investments to: develop modular, higher-throughput PBTK models; rapidly collect critical exposure and kinetic data; and transparently open access to both available models and associated data.

Generalizable building blocks for screening-level PBTK modeling of internal exposure will enable binning of data-poor chemicals for further mechanistic study and modeling. In vitro methods for experimentally determining some aspects of TK allow for more rapid development of TK models. These models can be simple [5] when based on in vitro data and steady-state assumptions, or full PBTK models [50] when combined with methods for predicting chemical concentration into tissues (i.e., partitioning [51]. While it is expected that a rapidly parameterized TK model may not perform as well as a tailored model developed with in vivo data, these do offer three significant advantages. First, the rapid model serves as a prototype for future models. If a confidence in the model can be predicted [50], then it may be either that the model is good enough as is or, depending on the application, it may be expanded to address specific needs. Second, the development of many rapid models in a systematic fashion allows methodical investigation of TK impacts, as a function of chemistry. In this way, trends may be elucidated to inform general TK model development. This approach can then be combined with appropriate portal-of-entry models based on potential routes of exposure and the physical-chemical properties of the environmental contaminants. Third, a generic model implementation that has been thoroughly tested is less likely to suffer from implementation and documentation errors that occur when reporting novel PBTK models [6, 7, 52, 53].

**Fig. 2:**
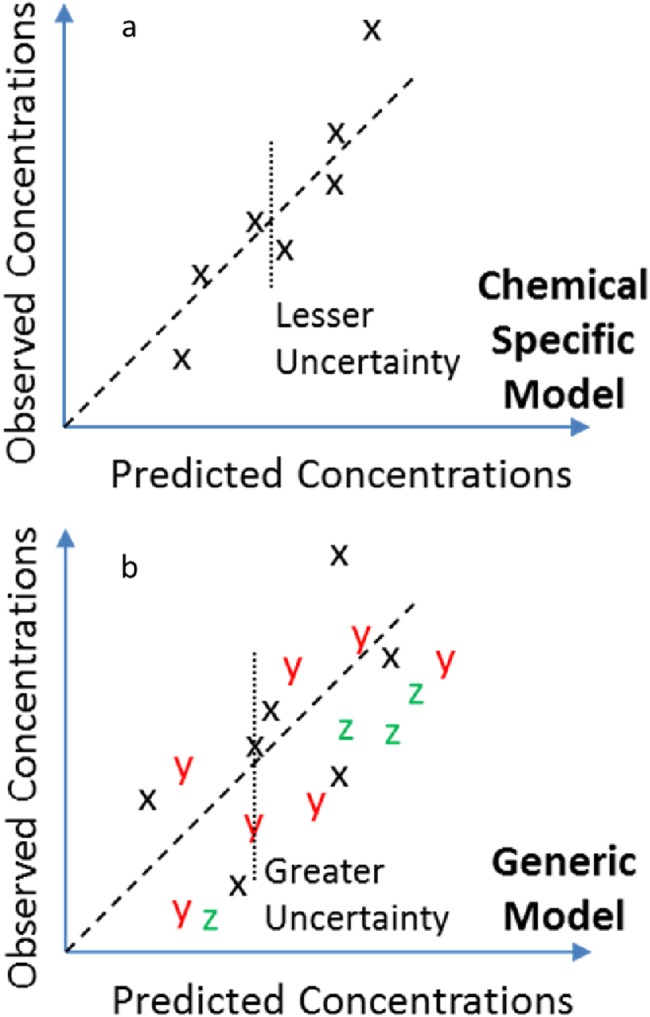
To evaluate a chemical-specific PBTK model for “chemical x” **a**, the predictions are compared to in vivo measured data for that chemical. For situations where chemical-specific TK data are not available** b**, generic TK models offer an alternative framework in which the model is parameterized and evaluated for all chemicals with in vivo data  and then extended for use with data poor chemicals

In order to address the large numbers of data-poor chemicals efficiently, recent research efforts have developed generic (as opposed to chemical-specific) screening level PBTK modeling approaches that use in vitro ADME data. When in vivo measured TK data (that is, chemical concentration vs. time) are available, these data allow evaluation of a chemical specific PBTK model. The predictions of the chemical specific model may be assessed for both bias and uncertainty [7], as illustrated in Fig. 2a. When no in vivo TK data are available, a generic PBTK model can instead be parmeterized [54–57]. To evaluate that model, overall predictions can be compared to in vivo TK data for those chemicals with data, as illustrated in Fig. 2b. Although predictions generated for any one chemical using a generic model can be expected to have larger uncertainty than those from a chemical-specific model, there can be greater confidence that the model structure has been appropriately implemented since it has been evaluated against a larger amount of data [6, 17].

Generic TK approaches amenable to high-throughput testing have been applied to assess risk indicated by toxological screening data [25, 58, 59]. The approaches were then refined to address the most significant uncertainties and evaluated to define chemical domain for which this type of approach was sufficiently resolved [60, 50, 61]. Just as chemical-specific models allow extrapolation to different exposure scenarios, a generic TK or PBTK model allows extrapolation to data-poor chemicals. It is therefore possible to correlate errors in the predictions of a generic PBTK model with chemical-specific properties [62, 50]. The initial generic PBTK models focused on volatile, non-ionizable chemicals [56], but models are now available to address non-volatile and ionized compounds [57].

In addition to a suite of generic PBTK model structures, direct access to physicochemical and ADME data is needed to make chemical-specific predictions. Historically, ADME data have been collected through experimental means, generally using targeted analytical chemistry methods. The prerequisites for targeted ADME studies include: (1) knowledge of the parent compound and related metabolites; (2) standards of all compounds to be analyzed; and (3) proven quantitative methods with acceptable levels of accuracy and precision. In light of these requirements, the number of compounds with quantitative TK data is limited. As such, advances in methods to measure and estimate model parameters will support efficient application of complex PBTK models, in addition to the new modular PBTK tools.

First tier estimates for PBTK parameters will leverage cheminformatics tools. Zang et al. [63] have generated an open-source quantitative structure–property relationship (QSPR) workflow to predict a variety of physicochemical properties that would have cross-platform compatibility to integrate into existing cheminformatics workflows, including those for estimating ADME parameters. Importantly, analytical techniques are also evolving in a manner that will enable the efficient collection of data to support TK model development, evaluation, and refinement. Two emerging approaches, known as suspect screening analysis (SSA) and non-targeted analysis (NTA), are largely based on high-resolution mass spectrometry (HRMS), and offer means to identify, and in some cases quantify, sample constituents with limited or no a priori information [64, 65]. Briefly, SSA are methods in which observed but unknown features are compared against a database of chemical suspects to identify plausible hits. NTA are those in which chemical structures of unknown compounds are postulated without the aid of suspect lists. These tools therefore hold great promise for expanding coverage of TK models across compounds with little or no existing data.

Compound identification in SSA is made possible through comparison of HRMS data against chemical screening lists (for examples, see: https://comptox.epa.gov/dashboard/chemical_lists). Screening lists can vary dramatically in size (tens to thousands of compounds) and include known parent compounds, as well as previously observed or model predicted metabolites [66]. Compound identification in NTA, however, proceeds without the use of screening libraries. Rather, HRMS features of interest are first selected (e.g., via statistical comparisons of control vs. treatment groups), with formulas and structures then proposed with the aid of supporting experimental data. SSA has a significant advantage over NTA in terms of throughput. However, feature annotation in SSA is restricted to compounds included on screening lists—i.e., those known or postulated to exist. NTA is decidedly lower throughput, but can be used to discover compounds never before studied. The combination of both methods, then, is a powerful approach for exploring chemical space in samples of interest that are understudied or completely unknown.

SSA and NTA can aid TK studies on two fronts: namely, hypothesis generation and model evaluation. At the most basic level of hypothesis generation, SSA/NTA studies can identify novel parent compounds for which toxicity, exposure, and TK data do not exist [67, 68]. A wealth of environmental and biological media have now been evaluated using SSA/NTA methods, and hundreds to thousands of never-before-studied compounds identified [69, 70]. After initial characterization, these novel compounds, as well as noteworthy mixtures, can be prioritized for exposure, bioactivity, and TK screening using QSAR-based methods [71]. Here, lower-tier TK models are the focus of initial investigations attempting to bridge any new exposure and toxicity information.

With respect to analyses of compounds that are known (i.e., those that have been previously studied and for which some exposure, toxicology, or TK data exist), SSA/NTA methods can be used to screen for derivative analytes that may be formed by metabolic processes and readily measured in biological samples [72, 73]. Here, metabolites of known parent compounds are identified via SSA/NTA, and measurement data used to generate and/or test hypotheses related to ADME processes. These data can inform the extent to which higher-tier TK models must address clearance through a specific pathway, or activation via a specific metabolic intermediate.

Uses of SSA/NTA methods for model evaluation are geared towards known but data-poor compounds for which only provisional estimates exist of steady-state blood, urine, or tissue concentrations. SSA methods, in particular, can be rapidly deployed to simultaneously collect surveillance data on thousands of compounds of interest. These methods can further provide initial concentration estimates for screened analytes using reference standardization techniques [74]. While these measures may lack the accuracy and precision of those based on targeted MS methods, they provide an initial means to evaluate high-throughput toxicokinetic (HTTK) model estimates. When warranted, SSA methods can be optimized, or targeted MS methods developed, to provide increased confidence in laboratory measures [71]. The primary goal of this evaluation step is to determine for which compounds higher-tier TK models are needed. A secondary goal is to inform the types of targeted measurement data that are required for model refinement.

Open-source access to evolving PBTK data and modeling tools is critical to ensure transparency in all risk-informed decision processes. Access to the information is necessary to evaluate the quality of the data, reproducibility of the modeling results, as well as to identify gaps in the analysis requiring further data and model development. With this in mind, scientists have devised a multi-pronged approach to provide access to valuable resources for the risk assessment community. Current efforts are focused on the development of an open-source platform to perform IVIVE and PBTK modeling for applications of varying complexity [57]. In addition, this platform will include: tools to assess population variability drawn from a range of U.S. populations; links to all publically available in vitro and in vivo TK data; tools trained on environmental chemicals to predict plasma protein binding [61] and in vitro hepatic clearance; and additional tools in development to address specific life-stages and sensitive populations [32].

Application of PBTK and internal exposure modeling tools will better support public health protective decisions as scientifically sound alternative approaches facilitate a shift away from reliance on defaults, and toward advanced technologies that allow holistic evaluation of chemicals. In addition, as more information about chemical kinetics is developed and incorporated in chemical evaluation workflows, uncertainties in alternative experimental systems will decrease [75]. Finally, as these new approaches and tools are evaluated and demonstrated through problem-driven application, the value added in a decision context will be tangible and quantifiable.

## Disclaimer

The U.S. EPA provided administrative review and approved this paper for publication. The views expressed in this paper are those of the authors and do not necessarily reflect the views of the U.S. EPA.
